# Draft Genome Sequence of *Thiohalobacter thiocyanaticus* Strain FOKN1, a Neutrophilic Halophile Capable of Thiocyanate Degradation

**DOI:** 10.1128/genomeA.00799-17

**Published:** 2017-08-10

**Authors:** Mamoru Oshiki, Toshikazu Fukushima, Shuichi Kawano, Junichi Nakagawa

**Affiliations:** a Department of Civil Engineering, National Institute of Technology, Nagaoka College, Nagaoka, Japan; b Advanced Technology Research Laboratories, Research & Development, Nippon Steel & Sumitomo Metal Corporation, Futtsu, Japan; c Department of Computer and Network Engineering, Graduate School of Informatics and Engineering, The University of Electro-Communications, Tokyo, Japan

## Abstract

A draft genome sequence of a neutrophilic halophile capable of thiocyanate degradation, *Thiohalobacter thiocyanaticus* FOKN1, was determined using a PacBio RSII sequencer. A 3.23-Mb circular genome sequence was assembled, in which 3,026 gene-coding sequences, 45 tRNAs, and 1 *rrn* operon were annotated.

## GENOME ANNOUNCEMENT

Thiocyanate is a major component of wastewater discharged from factories engaged in coal gasification (1). Removal of thiocyanate has been carried out using a biological procedure, such as an activated-sludge process (2), in which certain bacteria degrade thiocyanate aerobically to sulfate, carbon dioxide, and ammonia (3). So far, the thiocyanate-degrading freshwater bacterium *Thiobacillus thioparus* (4), neutrophilic halophile *Thiohalobacter thiocyanaticus* (5), and alkaliphilic halophile *Thioalkalivibrio* (6) have been isolated from activated sludge and soda and hypersaline lakes. Recently, genome sequences of a freshwater isolate and alkaliphilic halophiles were determined (7, 8). Here, we report the first draft genome sequence of a thiocyanate-degrading neutrophilic halophile.

We initially enriched thiocyanate-degrading neutrophilic halophiles from activated sludge by inoculating the biomass into an inorganic medium containing thiocyanate (per liter, 320 mg of sodium thiocyanate, 1,650 mg of ammonium chloride, 650 mg of sodium hydrogen carbonate, 29.2 mg of disodium phosphate, 0.001% [wt/vol] yeast extract, and 60% [vol/vol] seawater [pH 7.0 to 7.5]). The culture was aerobically incubated at 30°C, and a thiocyanate-degrading neutrophilic halophile strain, FOKN1, was isolated from the enrichment culture by the serial dilution method. Strain FOKN1 degraded 200 mg/liter thiocyanate within 1 week and produced ammonium in culture quantitatively. The isolated cells were morphologically uniform, and a monospecies culture of strain FOKN1 was ascertained by determining 16S rRNA gene sequences, as described elsewhere (9).

Genomic DNA was extracted using the Qiagen DNeasy minikit (Qiagen, Tokyo, Japan) and was subjected to sequencing on a PacBio RSII sequencer and single-molecule real-time (SMRT) cell 8Pac V3, with the DNA polymerase binding kit P6 (Pacific Biosciences, CA). The sequencing produced 146,654 valid reads (average length, 7,905 bp), which were assembled into a 3.23-Mb circular genomic sequence by means of the HGAP3 software (10). Gene prediction and annotations were performed via the MiGap pipeline (11), as previously mentioned (12), and 3,026 coding sequences (CDSs), 1 *rrn* operon, and 45 tRNA genes were annotated. The full-length 16S rRNA gene sequence (1,504 bp) from the genome was subjected to BLASTn analysis, and the 16S rRNA gene sequence showed 99.1% similarity with that of the thiocyanate-degrading neutrophilic halophile *Thiohalobacter thiocyanaticus* HRh1, previously isolated from hypersaline chloride-sulfate lakes (5). Because 99% similarity of the 16S rRNA gene sequence has been employed as a threshold above which a bacterium can be assigned to the same bacterial species (13), the strain FOKN1 was here designated *Thiohalobacter thiocyanaticus* strain FOKN1.

A gene encoding thiocyanate dehydrogenase was present in the *Thiohalobacter thiocyanaticus* FOKN1 genome as well as the *Thioalkalivibrio thiocyanoxidans* ARh2 genome (GenBank accession number ARQK00000000), while a gene encoding thiocyanate hydrolase was missing (7). This finding suggests that FOKN1 cells degrade thiocyanate by using thiocyanate dehydrogenase instead of thiocyanate hydrolase, which should be further investigated.

### Accession number(s).

The *Thiohalobacter thiocyanaticus* FOKN1 genome was deposited in the DDBJ nucleic acid sequence database under the GenBank accession no. AP018052.
